# Optimizing the Energy Harvesting Cycle of a Dissipative Dielectric Elastomer Generator for Performance Improvement

**DOI:** 10.3390/polym10121341

**Published:** 2018-12-04

**Authors:** Peng Fan, Hualing Chen

**Affiliations:** 1 State Key Laboratory for Strength and Vibration of Mechanical Structures, Xi’an Jiaotong University, Xi’an 710049, China; fpwendong@gmail.com; 2 School of Mechanical Engineering, Xi’an Jiaotong University, Xi’an 710049, China

**Keywords:** electroactive polymer, dielectric elastomer, energy harvesting, loss of tension, leakage current

## Abstract

This paper optimizes the energy harvesting cycle of dissipative dielectric elastomer generators (DEGs) to explore possible approaches for improving the energy harvesting performance. By utilizing the developed theoretical framework, the dissipative performance of the DEG with a constant voltage cycle is analyzed, which shows good agreement with the existing experimental data. On this basis, we design a novel energy harvesting cycle and a corresponding energy harvesting circuit in which a transfer capacitor is utilized to store the charge transferred from the DEG. Then, the energy conversion performance of the DEG with the novel energy harvesting cycle is investigated. The results indicate that both the energy density and conversion efficiency are improved by choosing a high voltage during the discharging process and that as the R-C time constant increases, the enhancement effect of the voltage increases and then approaches to the saturation. In addition, there is an optimal transfer capacitor that can maximize energy density or conversion efficiency, and the optimal transfer capacitor increases with the increase in the R-C time constant. These results and methods are expected to guide the optimal design and assessment of DEGs.

## 1. Introduction

As a category of electroactive polymers, dielectric elastomers (DEs) are attractive smart materials due to intrinsic attributes, such as high energy density, lightweight, flexibility, and large deformation [1,2,3,4,5,6]. A dielectric elastomer generator (DEG) can harvest electrical energy from diverse mechanical energy sources, including flowing water [7], ocean waves [7,8,9,10], wind [11], and human motion [6,12,13,14]. A DEG consists of a soft DE membrane, sandwiched between two compliant electrodes. A typical energy harvesting cycle is summarized as follows. The DEG can be first stretched by the external mechanical forces and then charged by the power supply; then, the DEG is mechanically contracted under the open circuit condition, which boosts the voltage between the electrodes, accomplishing the charge transfer from a low potential to a high potential; the charge at a high potential is harvested before the beginning of the next cycle. 

Since Pelrine et al. first proposed a DEG embedded in a heel of a shoe [6], many DEGs with different applications and energy harvesting cycles have been developed. Some researchers focused on how to use DEGs in practical applications. For example, to develop wearable DEGs, Jean-Mistral et al. proposed a DEG polarized by an electret instead of a power supply [14], and McKay et al. developed a DEG with a self-priming circuit used as the inverse charge pump [15]. To convert wave energy into electrical energy, Moretti et al. proposed a DEG-based wave energy converter that consists of a circular diaphragm DEG and a hydrodynamic interface [16], and Chiba et al. developed a DEG embedded on a buoy [17]. Some studies aimed at improving the energy density of the DEG by designing and optimizing the energy harvesting cycle. Fan et al. enhanced the energy density of a DEG by reducing the pre-stretch ratio and enlarging the cycle period [18]. Zhou et al. achieved a high energy density by choosing an appropriate stretch ratio for the onset of the discharging process [19]. Foo et al. improved the energy density of a dissipative DEG by increasing the maximum stretch ratio and extending the cycle period [20]. Shian et al. utilized the loss of tension (LT) process, i.e., the relaxation process under the condition of no external mechanical force, as the discharging process to optimize the energy harvesting cycle, which achieved experimentally an energy density of 0.78 J/g [21]. By comparing with the DEG embedded in a heel (energy density of 0.3 J/g), the energy density of DEGs was significantly improved, but it is still lower than the theoretical maximum energy density of 1.7 J/g [22]. Moreover, improving the energy density by using the LT process as the discharging process is at the cost of decreasing the conversion efficiency of the DEG. It is reported that enlarging the deformation rate during the relaxing process can improve both energy density and conversion efficiency [5]. However, compared with the experimental results reported in the literature [21], the conversion efficiency is not significantly improved. The main reason for this is the energy dissipation caused by the DE material viscosity, especially during the LT process. Therefore, it is interesting to explore the feasible approaches for improving both energy density and conversion efficiency by designing and optimizing the energy harvesting cycle of DEGs, aiming at achieving significant performance improvement. As is well known, DEs in general possess viscoelastic properties like other polymeric materials. For describing the viscoelastic behavior, Hong’ viscoelastic model [23] is representative. For example, utilizing Hong’ viscoelstic model and the constant voltage cycle, Foo et al. investigated the performance of a dissipative DEG by considering the material viscosity and the leakage current [20]; Li et al. analyzed the performance of viscoelastic DEGs under the inhomogeneous deformation [24]; Zhou et al. studied the energy conversion of viscoelastic DEGs with consideration of the fatigue life [25], and Chen et al. investigated the effect of temperature on dissipative DEGs with the material failure modes [26].

This paper aims to explore possible approaches for significantly improving both energy density and conversion efficiency. First, a theoretical framework is proposed by considering the material viscosity and the leakage current. Then, to validate the developed theoretical framework, the dissipative performance of the DEG with the constant voltage cycle is studied, which is consistent with existing experimental results. On this basis, we design a novel energy harvesting cycle and a corresponding energy harvesting circuit in which a transfer capacitor is utilized to store the charge transferred from the DEG, and then investigate the influence of the transfer capacitor, the voltage during the discharging process, and the R-C time constant of the elastomer on the energy harvesting performance of a dissipative DEG. The results and methods can guide the optimal design and assessment of DEGs.

## 2. Theoretical Framework for Dissipative DEGs

**Figure 1:**
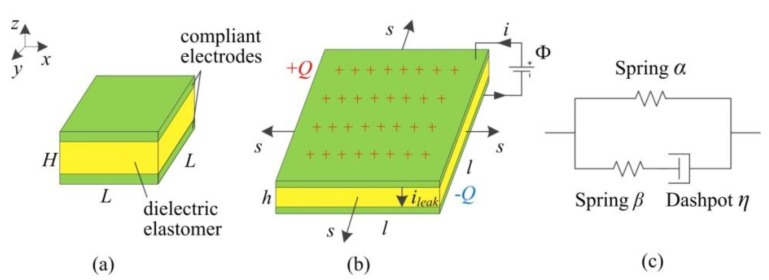
(**a**) A DEG in the reference state. (**b**) In a stretched and charged state, subjected to an equal-biaxial stress, *s*, and a voltage, Φ, the DEG is charged and stretched, accompanied by a leakage current, *i*_leak_, in the thickness direction. (**c**) The standard linear solid (SLS) viscoelastic model.

As shown in Figure 1a, a DEG is in the reference state with the lengths, *L* and *L*, and the thickness, *H*. When the membrane is subjected to an equal-biaxial stress, *s*, and a voltage, Φ, each electrode gets a charge, *Q*, and the membrane deforms homogeneously to the state with the lengths, *l* and *l*, and the thickness, *h*, accompanied by a leakage current, *i*_leak_, in the thickness direction, as shown in Figure 1b. The stretch ratio is defined as *λ* = *l*/*L*. The membrane is considered incompressible, namely, *h* = *Hλ*^−2^. 

Considering the current leakage, the DEG is represented by a capacitor in parallel with a conductor [2]. The capacitance of the DEG capacitor is *C* = *ε*_0_*ε*_r_*L*^2^*H*^−1^*λ*^4^, where *ε*_0_ is the vacuum permittivity, and *ε*_r_ is the relative dielectric constant of the DE material. As a capacitor, the charge on the DEG can be expressed as *Q* = *C*Φ. In addition, the electrodes and the wires are regarded as the perfect conductors. As shown in Figure 1b, the current in the conducting wire attached to the electrodes is expressed as *I* = d*Q*/d*t* + *i*_leak_. The leakage current can be written as follows [2]:

(1) $$i_{\mathrm{leak}}=\sigma_{c0}\frac{\Phi L^{2}\lambda^{4}}{H}\exp\left(\frac{E}{E_{B}}\right),$$

where *E* = Φ*λ*^2^/*H* is the electric field, *σ_c_*_0_ is the conductivity in a low electric field, and *E_B_* is an empirical constant.

As shown in Figure 1c, the standard linear solid (SLS) viscoelastic model is adopted to characterize the viscoelastic behavior [2,20,23]. In the SLS model, one unit consists of a hyperelastic element, *α*, and the other unit consists of a hyperelastic element, *β*, and a series-wound dashpot. By utilizing the SLS model, the stretch ratio, *λ*, can be regarded as the stretch ratio of both units. Thus, the element, *α*, can deform by the stretch ratio, *λ*. The stretch ratio of the element, *β*, is characterized by the stretch ratio, *λ*^i^, which is determined by the multiplication rule as *λ*^i^ = *λ*/*ξ* [2,20,23], where *ξ* is the corresponding inelastic stretch ratio in the dashpot. To describe the strain-stiffening effect of DEs, the Gent model [27] is used. Based on the thermodynamics theories and assumptions [2], the governing equation of viscoelastic DEGs is expressed as:

(2) $$\frac{\chi \left(\lambda -\lambda^{-5}\right)}{1-\left(2\lambda^{2}+\lambda^{-4}-3\right)/J^{\alpha }}+\frac{\left(1-\chi \right)\left(\lambda \xi^{-2}-\lambda^{-5}\xi^{4}\right)}{1-\left(2\lambda^{2}\xi^{-2}+\lambda^{-4}\xi^{4}-3\right)/J^{\beta }}-\frac{\varepsilon_{0}\varepsilon_{r}\Phi^{2}}{\mu H^{2}}\lambda^{3}-\frac{s}{\mu }=0,$$

where *μ =* (*μ^α^* + *μ^β^*) denotes the instantaneous shear modulus of the DE, *μ^α^* and *μ^β^* are the shear modulus of the element, *α*, and the element, *β*, *J^α^* and *J^β^* are the material constants related to the limiting stretch ratios of the two elements, and *χ* = *μ^α^*/*μ* is the fraction of the polymer networks that have the time-independent behavior [5,25,28]. The response of the dashpot has the same constitutive model as a Newtonian fluid, and the deformation rate in the dashpot is described as *ξ*^−1^d*ξ*/d*t* and can be related to the stress as [2,20]:

(3) $$\frac{d\xi }{dt}=\frac{\mu^{\beta }}{6\eta }\frac{\lambda^{2}\xi^{-1}-\lambda^{-4}\xi^{5}}{1-\left(2\lambda^{2}\xi^{-2}+\lambda^{-4}\xi^{4}-3\right)/J^{\beta }},$$

where *η* is the viscosity of the dashpot. The viscoelastic relaxation time is defined as *t*_v_ = *η*/*μ^β^*. 

In an energy harvesting cycle, as the external mechanical force applied to the membrane decreases, the membrane can relax. When the stress decreases to zero, the condition is known as LT. From the existing experiments [21,29], it is observed that the viscoelastic membrane can continue to relax under the condition of no stress, which is referred to as the LT process. In the LT process, a large amount of energy is dissipated due to the material viscosity, which can affect significantly the energy harvesting performance of DEGs. When the equal-biaxial stress, *s* = 0, Equation (2) can be written as an implicit function, F (*λ*, *ξ*, Φ) = 0, where:

(4) $$F(\lambda ,\xi ,\Phi )=\frac{\chi \left(\lambda -\lambda^{-5}\right)}{1-\left(2\lambda^{2}+\lambda^{-4}-3\right)/J^{\alpha }}+\frac{\left(1-\chi \right)\left(\lambda \xi^{-2}-\lambda^{-5}\xi^{4}\right)}{1-\left(2\lambda^{2}\xi^{-2}+\lambda^{-4}\xi^{4}-3\right)/J^{\beta }}-\frac{\varepsilon_{0}\varepsilon_{r}\Phi^{2}}{\mu H^{2}}\lambda^{3}.$$

Therefore, during the LT process, the deformation rate of the DE can be expressed as [5]:

(5) $$\frac{d\lambda }{dt}=\frac{d\lambda }{d\xi }\frac{d\xi }{dt}=-\frac{F_{\xi }}{F_{\lambda }}\frac{d\xi }{dt}=-\frac{\mu^{\beta }}{6\eta }\frac{\lambda^{2}\xi^{-1}-\lambda^{-4}\xi^{5}}{1-\left(2\lambda^{2}\xi^{-2}+\lambda^{-4}\xi^{4}-3\right)/J^{\beta }}\frac{F_{\xi }}{F_{\lambda }}.$$

where F_λ_ and F_ξ_ are the differential of the function, F (*λ*, *ξ*, Φ), with respect to *λ* and *ξ*, respectively, and thus:

(6) $$F_{\xi }=\frac{-\left(1-\chi \right)\left(2\lambda^{-2}\xi^{-3}+4\lambda^{-8}\xi^{3}\right)}{1-\left(2\lambda^{2}\xi^{-2}+\lambda^{-4}\xi^{4}-3\right)/J^{\beta }}+\frac{-4\left(1-\chi \right)\lambda^{4}\xi^{-1}{\left(\lambda^{-2}\xi^{-2}-\lambda^{-8}\xi^{4}\right)}^{2}/J^{\beta }}{{\left(1-\left(2\lambda^{2}\xi^{-2}+\lambda^{-4}\xi^{4}-3\right)/J^{\beta }\right)}^{2}}$$

and:

(7) $$\begin{array}{ll} F_{\lambda } & =\frac{-2\chi \left(\lambda^{-3}-4\lambda^{-9}\right)}{1-\left(2\lambda^{2}+\lambda^{-4}-3\right)/J^{\alpha }}+\frac{4\chi \lambda^{3}{\left(\lambda^{-2}-\lambda^{-8}\right)}^{2}/J^{\alpha }}{{\left(1-\left(2\lambda^{2}+\lambda^{-4}-3\right)/J^{\alpha }\right)}^{2}}\\ & +\frac{-2\left(1-\chi \right)\left(\lambda^{-3}\xi^{-2}-4\lambda^{-9}\xi^{4}\right)}{1-\left(2\lambda^{2}\xi^{-2}+\lambda^{-4}\xi^{4}-3\right)/J^{\beta }}+\frac{-4\left(1-\chi \right)\lambda^{3}{\left(\lambda^{-2}\xi^{-2}-\lambda^{-8}\xi^{4}\right)}^{2}/J^{\beta }}{{\left(1-\left(2\lambda^{2}\xi^{-2}+\lambda^{-4}\xi^{4}-3\right)/J^{\beta }\right)}^{2}} \end{array}$$

In an electromechanical cycle, the dimensionless dissipated energy due to the leakage current *W*_leak_ is as follows:

(8) $$W_{\mathrm{leak}}=\frac{1}{L^{2}H\mu }\int \Phi i_{\mathrm{leak}}dt.$$

The dimensionless electrical energy density generated in a cycle, *E*_density_, is as follows:

(9) $$E_{\mathrm{density}}=-\frac{1}{L^{2}H\mu }\int \Phi idt.$$

The input dimensionless mechanical energy in a cycle, *W*_mech_, is expressed as follows:

(10) $$W_{\mathrm{mech}}=\frac{2}{\mu }\int sd\lambda .$$

The electromechanical conversion efficiency of a DEG is written as follows:

(11) $$\eta_{c}=E_{\mathrm{density}}/W_{\mathrm{mech}}.$$

## 3. Evaluating the Theoretical Framework

**Figure 2:**
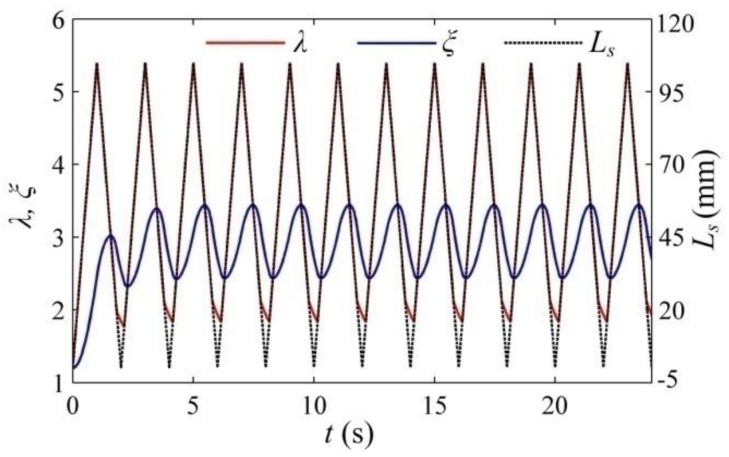
The deformation of the DEG and the variation of the servo displacement for the first twelve cycles.

**Figure 3:**
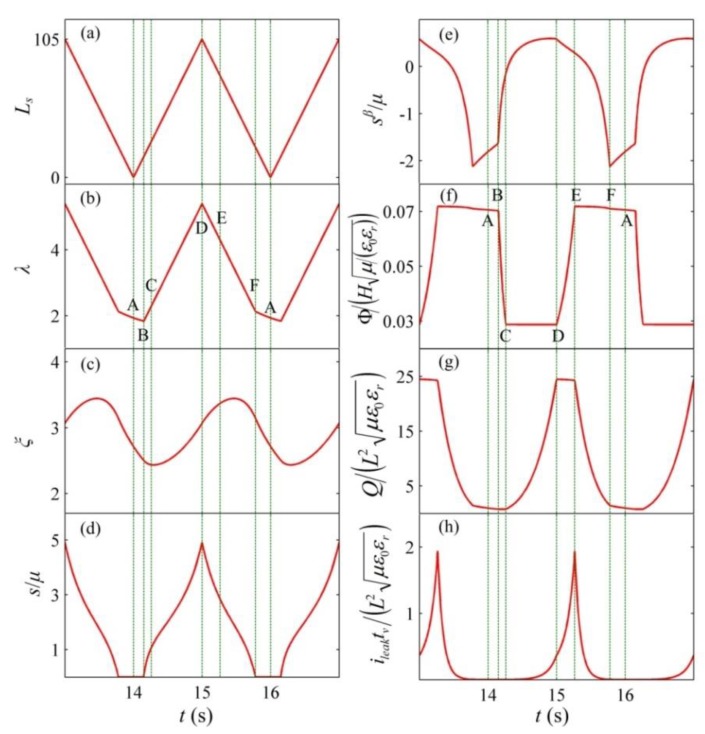
Variables as functions of time in a steady cycle. (**a**) The stretch ratio, (**b**) the inelastic stretch ratio, (**c**) the dimensionless nominal stress of the DE, (**d**) the dimensionless voltage, (**e**) the dimensionless charge, and (**f**) the dimensionless leakage current. (**g**) the dimensionless charge, and (**h**) the dimensionless leakage current. *s^β^* is the stress on the dashpot, which is equal to the stress acting on the element, *β*.

**Figure 4:**
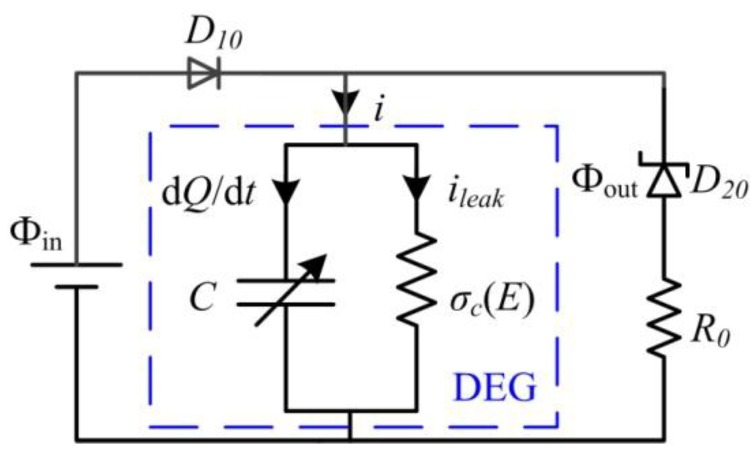
Energy harvesting circuit diagram of the DEG with the constant voltage cycle.

In the following section, by employing the established theoretical framework, we investigate the energy conversion of a dissipative DEG with the constant voltage cycle in which it can relax during the LT process. To use the existing experimental data [29] to evaluate the established framework, in this simulation, the same VHB 4905 is chosen as the DE material and the material parameters are summarized in Table 1. The geometrical parameters of the DEG are also the same as those reported in the literature [29] and selected as *H* = 0.5 mm, the minimum stretch ratio as *λ*_min_ = 1.2, and the maximum stretch ratio as *λ*_max_ = 5.4. In the experiment [29], the VHB-based DEG was loaded by the motion of a linear servo motor, which is simulated in this section. According to the literature [29], the servo displacement, *L_s_*, ranges from 0 to 105 mm. Figure 2 describes the deformation of the DEG and the variation of the servo displacement for the first 12 cycles. The simulation results indicate that the DEG reaches a steady state after a few cycles, which is similar with the conclusion reported by the literature [20]. Figure 3 depicts the response of the DEG in a steady cycle. The parameter, *s^β^*, denotes the stress on the dashpot, which is equal to the stress acting on the element, *β*. Figure 4 shows the energy harvesting circuit of the DEG with the constant voltage cycle. For consistency with the experiment [29], the input voltage of the power supply is Φ_in_ = 2000 V, the reverse breakdown voltage of the zener diode is *D_20_* Φ_out_ = 5000 V, and the limiting resistors is *R*_0_ = 50 kΩ. The implementation process of the constant voltage cycle is summarized as follows. At state A, though the servo loading cycle starts, the DEG does not start to be stretched and is still in the relaxing process. When the servo motor moves to state B, the DEG starts to be stretched by the servo motor. During interval BC, as the stretch ratio increases, the voltage between the electrodes decreases from Φ_out_ to Φ_in_. During this process, the membrane is stretched under the open circuit condition and the charge on the electrodes can be dissipated slightly due to the leakage current. After state C is reached, the diode, *D*_10_, conducts and the DEG is charged until it is stretched to the maximum stretch ratio, *λ*_max_ (state D). During interval CD, the voltage remains constant. During the stretching process (form state B to D), the membrane is mechanically stretched by the servo motor at a deforming rate of d*λ*/d*t* = 4.2 s^−1^. At state D, the membrane stops being stretched and starts to be relaxed. As the stretch ratio decreases, the voltage can increase gradually. When the voltage increases to Φ_out_ at state E, the reverse breakdown of the zener diodes, *D*_20_, occurs. From state D to E, the DEG is relaxed under the open circuit condition and the charge on the electrodes can be dissipated slightly due to the leakage current. When state E is reached, the DEG starts to be discharged. Then, the membrane continues to be relaxed until LT appears (the external force decreases to zero) at state F. In interval DF, the membrane is mechanically relaxed at a deforming rate of d*λ*/d*t* = −4.2 s^−1^. After state F is reached, the membrane can further contract during the LT process. When the membrane reaches state A, the servo motor returns to the initial position and the servo loading cycle, ABCDEFA, is completed. Then, the membrane continues to relax during the LT process until it reaches state B of the next servo loading cycle. In interval FB, the membrane operates during the LT process and the deforming rate of the membrane meets Equation (5). During the discharging process (from state E to B), the voltage remains constant. After the membrane reaches state B, the membrane starts to be stretched again, i.e., the DEG starts to execute the next energy harvesting cycle, BCDEFAB. Therefore, there is a delay between the servo loading cycle and the energy harvesting cycle, which is consistent with the experimental observations [29].

**Figure 5:**
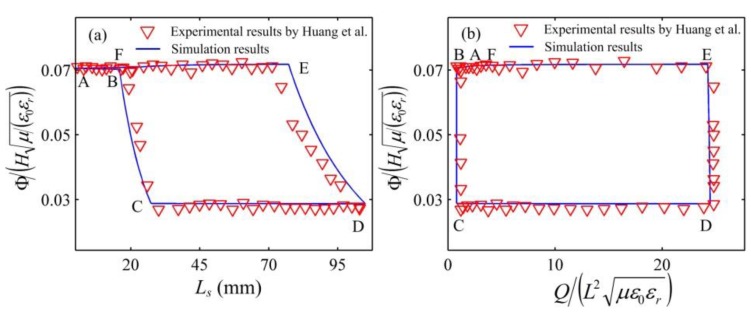
Voltage between the electrodes (**a**) as a function of the servo displacement and (**b**) as a function of the charge in the fifth cycle.

**Figure 6:**
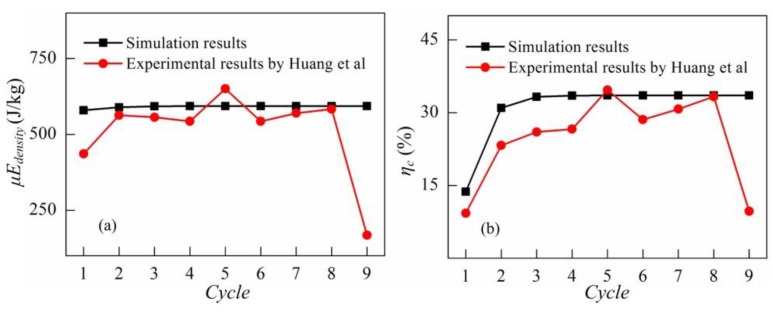
Energy conversion of the DEG for the first nine cycles. (**a**) The energy density and (**b**) the conversion efficiency.

The response of the DEG in the fifth cycle is plotted in Figure 5. It is noticed from Figure 5 that the constant voltage cycle in which the DEG can relax during the LT process is accurately described and the simulation results are consistent with the experimental data reported in the literature [29]. Figure 6 shows the energy density and the conversion efficiency for the first nine cycles. In our simulations, the average energy density is 591 J/g and the average conversion efficiency is 31%. The experimental results show the average energy density of 560 J/g and the average conversion efficiency of 27% [29]. The simulation results show good agreement with the experimental results reported by the literature [29]. The difference between the simulation results and the experimental results [29] might be due to the energy dissipation caused by energy harvesting circuits and friction in the experiment setup not being considered in our simulations. In the constant voltage cycle, when the external force decreases to zero, the DEG ends to be relaxed and starts to be stretched. This can prevent the DEG from relaxing during the LT process and make the conversion efficiency increase to 47% [28]. Therefore, preventing the DEG from relaxing during the LT process in an energy harvesting cycle is an effective method for improving the conversion efficiency. On the other hand, it is also indicated that the conversion efficiency can be significantly overestimated when the LT process in an energy harvesting cycle is neglected. 

## 4. Designing the Energy Harvesting Cycle

**Figure 7:**
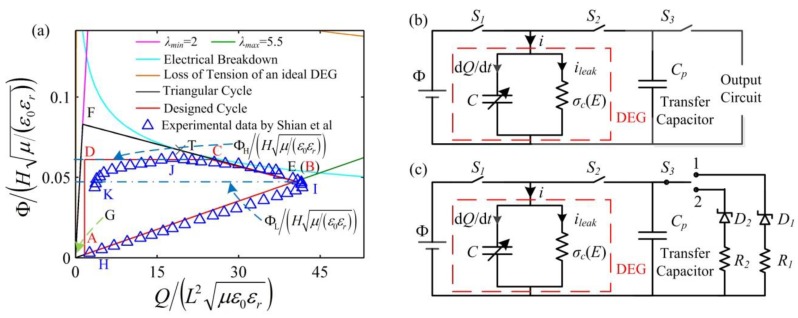
(**a**) The energy harvesting cycles; (**b**) the energy harvesting circuit diagram of the triangular cycle; and (**c**) the energy harvesting circuit diagram of the designed cycle.

In Figure 7a, the enclosed curve GEFG denotes the triangular energy harvesting cycle of an ideal DEG, i.e., a DEG without consideration of the material viscosity and the leakage current. Figure 7b shows the energy harvesting circuit of the triangular cycle and the capacitance of the transfer capacitor is *C*_p_. The implementation process of the triangular cycle has been well described in the literature [5,21] and is briefly summarized as follows. State G represents the stretching process in which the membrane is stretched from the prescribed minimum stretch ratio, *λ*_min_, to the maximum stretch ratio, *λ*_max_. At state G, the switches, *S*_1_, *S*_2_, and *S*_3_, are open. After the stretch ratio increases to *λ*_max_, the switches, *S*_1_ and *S*_2_, are closed. Then, the DEG is charged until the voltage between the electrodes increases to Φ_L_ at state E. After state E is reached, the switch, *S*_1_, is opened and the DEG starts to be relaxed. To maintain the same voltage level between the DE and the transfer capacitor, with the decrease in the stretch ratio, the charges on the DEG flow to the transfer capacitor. For an ideal DEG, the membrane can be relaxed from state E to F along the line, EF. The slope of the line, EF, in the dimensionless plane is −*C*_0_/*C*_p_, where *C*_0_ is the capacitance of the DE in the undeformed state. When state F is reached, the switch, *S*_3_, is closed to start the discharging process in which residual charges on the electrodes are harvested until the voltage on the DEG decreases to zero at state G. Then, the switches, *S*_2_ and *S*_3_, are opened and the membrane starts to be stretched again, i.e., the next cycle starts to be implemented. When the line, EF, is tangent to the electrical breakdown (EB) curve, the area enclosed by the triangle, EFG, reaches the maximum value. The coordinate of the tangency point, T, is (*x*_T_, *y*_T_). The EB voltage can be expressed as Φ_EB_ = *E_EB_Hλ*^(*R*−2)^, where *E*_EB_ is the electric breakdown field of the undeformed DE, and *R* is the strengthening exponent [22]. The relation between the capacitance, *C*_p_, and the voltage, Φ_L_, is expressed as follows [5]:

(12) $$\Phi_{L}=\frac{H\left(C_{p}y_{T}+C_{0}x_{T}\right)\sqrt{\varepsilon_{0}\varepsilon_{r}/\left(\chi \mu \right)}}{C_{p}+C_{0}\lambda_{max}^{4}},$$

where $y_{T}={\left(\frac{E_{EB}}{\sqrt{\varepsilon_{0}\varepsilon_{r}/\left(\chi \mu \right)}}\right)}^{4/\left(R+2\right)}x_{T}^{\left(R-2\right)/\left(R+2\right)}$ and $x_{T}=\frac{E_{EB}}{\sqrt{\varepsilon_{0}\varepsilon_{r}/\left(\chi \mu \right)}}{\left(\frac{C_{p}\left(2-R\right)}{C_{0}\left(2+R\right)}\right)}^{\left(R+2\right)/4}$.

To evaluate the above triangle cycle, in the literature [21], the experimental study is carried out. The experimental data are described in Figure 7a. It is found that the external force decreases to zero, i.e., LT appears, at state J before the DEG is relaxed to *λ*_min_, which results in the ending of the charge transfer between the DEG and the transfer capacitor. The main reason is that the actual DEG is with the viscosity and the leakage current and the LT of an actual DEG can be affected by the rate of deformation and the DEG dynamics, which is neglected in the simulation of the triangular cycle GEFG. Then, the discharging process (from state J to K) is implemented. In this process, the DEG relaxes during the LT process. In this experiment [21], the energy density of the DEG is up to 0.78 J/g, which is higher than those in other existing experiments. However, the conversion efficiency is low and about 30%. In the previous works [5], the implementation process of the experiment [21] is accurately simulated. The simulation results show that the reason for the low conversion efficiency is mainly that a large quantity of energy is dissipated during the LT process. Also, the conversion efficiency is not significantly improved by optimizing the energy harvesting cycle implemented in the experiment [21]. Therefore, a novel energy harvesting cycle is designed and optimized to further improve both the energy density and the conversion efficiency. Especially, the conversion efficiency can be significantly enhanced. Thus, in the designed energy harvesting cycle, the DEG is prevented from relaxing during the LT process, aiming at improving the conversion efficiency.

**Figure 8:**
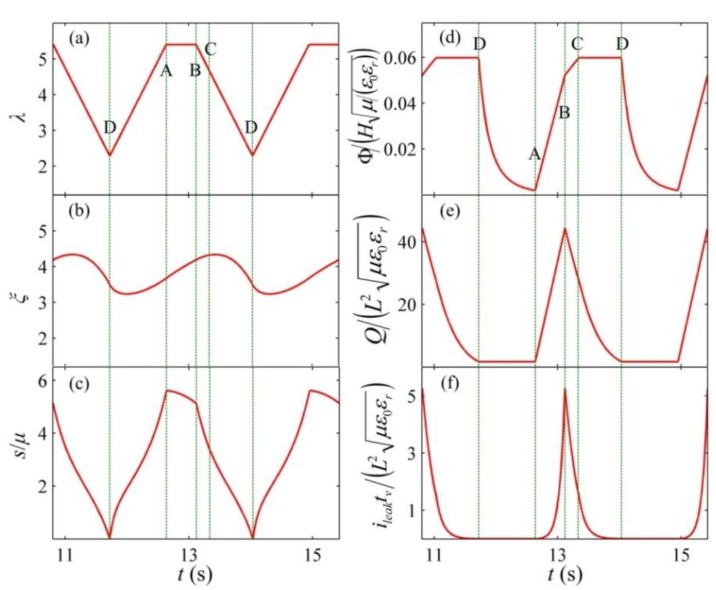
Variables as functions of time in a steady cycle. (**a**) The stretch ratio, (**b**) the inelastic stretch ratio, (**c**) the dimensionless nominal stress of the DE, (**d**) the dimensionless voltage, (**e**) the dimensionless charge, and (**f**) the dimensionless leakage current.

**Figure 9:**
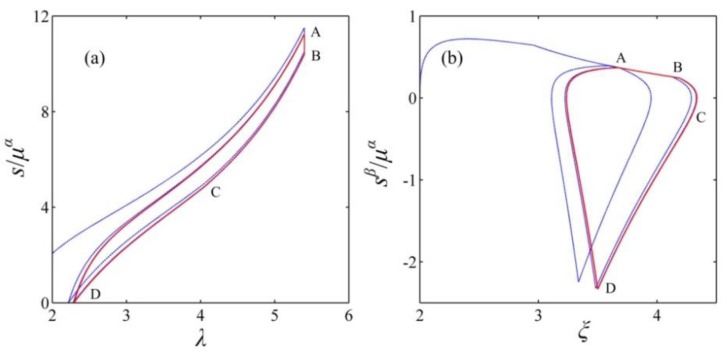
The energy harvesting cycle is represented in diagrams of work-conjugating variables. The blue dotted line describes the response from the start of the cycle, while the red solid line describes the steady cycle.

As shown in Figure 7a, the enclosed curve, ABCDA, denotes the designed energy harvesting cycle. The corresponding energy harvesting circuit is described in Figure 7c. The reverse breakdown voltage of the zener diodes, *D*_1_ and *D*_2_, is Φ_H_ and Φ_L_, respectively, and Φ_H_ > Φ_L_. The capacitance of the transfer capacitor is selected based on Equation (12). In addition, the designed energy harvesting circuit is only suitable for the purpose of experimental demonstration of the proposed cycle, not for real-world implementation. Figure 8 describes the response of the DEG in a cycle. The implementation process of the designed energy harvesting cycle is described as follows. At state A, switches 1 and 2 are open, and switch 3 is closed to position 1. Then, switches 1 and 2 are closed and the DE and the transfer capacitor start to be charged until the voltage between the electrodes increases to Φ_L_ at state B. When the DEG reaches state B, switch 1 is opened again and the charging process ends. Then, the membrane is relaxed, boosting the voltage between two electrodes due to the decrease in the capacitance of the DE. As the voltage increases, the charges on the electrodes flow to the transfer capacitor to maintain the same voltage level between the DE and the transfer capacitor. When the voltage between the electrodes increases to Φ_H_ (state C), the reverse breakdown of the zener diodes, *D_1_*, occurs, resulting in ending the charge transfer between the DE and the transfer capacitor. During interval BC, the current, *i* = d*Q*/d*t* + *i*_leak_ = −*C*_p_dΦ/d*t*. Then, the membrane can continue to be relaxed, and the charges on the electrodes flow to the zener diodes, *D*_1_, and the current limiting resistor, *R*_1_. When state D is reached, LT occurs. During the discharging process, namely, from state C to D, the voltage between the electrodes remains constant. To avoid more energy dissipation in the LT process, when LT occurs at state D, the DEG should end to be relaxed and discharged. Thus, after state D is reached, switch 2 is opened, and switch 3 is closed to position 2. Then, the membrane is stretched under the open circuit condition until the stretch ratio increases to *λ*_max_ at state A, and the voltage on the transfer capacitor can decrease to Φ_L_ due to the reverse breakdown voltage, Φ_L_, of the zener diodes, *D_2_*. When state A is reached, switches 1 and 2 are closed, and switch 3 is closed to position 1. Then, the DEG starts to be charged again. Figure 9 depicts that a closed loop is formed after a few cycles, namely, the DEG attain a steady state after several cycles. In the following section, we focus on the energy conversion of the DEG in a steady cycle.

In the literature [21], VHB is also selected as the DE material and the geometrical parameter, *H*, is the same as those reported in the literature [29]. However, values of the parameters, *λ*_min_ and *λ*_max_, are different from those referred to in the literature [29]. For comparison with the experimental results [21], in this simulation, the same DE material and geometrical parameters should be selected, and thus, *λ*_min_ = 2 and *λ*_max_ = 5.5 are set. According to the literature [22], *E*_EB_ = 30.6 MV/m and *R* = 1.13. The electrical parameters of the harvesting circuit are set as Φ_H_ = 4.4 kV, the current limiting resistors, *R*_1_ = *R*_2_ = 50 kΩ, and the capacitance ratio, $m=C_{p}/\left(C_{0}\lambda_{\max}^{4}\right)=1.3$. For consistency with the experiment reported in the literature [21], the membrane is stretched at a deformation rate of d*λ*/d*t* = 3.4 s^−1^ and relaxed at a deformation rate of d*λ*/d*t* = −3.4 s^−1^. During the charging process (from state A to B), the voltage between the electrodes increases at a rate of dΦ/d*t* = 2 Φ_L_. As shown in Figure 7a, the designed cycle, ABCDA, is consistent with the experimental data [21]. In our simulation, the energy density of the DEG with the designed cycle is 0.81 J/g, which is slightly higher than the experimental results [21] (0.78 J/g).

**Figure 10:**
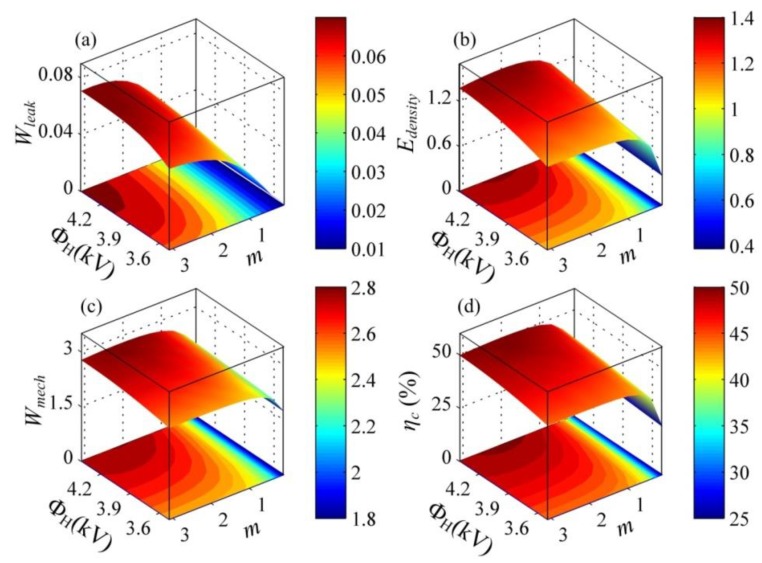
The influence of the voltage, Φ_H_, and the capacitance ratio, *m*, on the performance of a DEG. (**a**) The dimensionless dissipated energy due to leakage current, *W*_leak_, (**b**) the dimensionless energy density, *E*_density_, (**c**) the dimensionless mechanical energy, *W*_mech_, and (**d**) the conversion efficiency, *η*_c_.

According to the above analysis, in a steady energy harvesting cycle, the voltage, Φ_L_, at state B (which is related to the transfer capacitor) and the voltage, Φ_H_, at state C (which is determined by the zener diodes, *D*_1_) can affect the energy harvesting cycle. This is, the capacitance ratio, *m*, and the voltage, Φ_H_, during the discharging process can obviously influence the performance of DEGs. To provide better guidance for optimizing the proposed energy harvesting cycle, we investigate the effect of the capacitance ratio, *m*, and the voltage, Φ_H_, on the performance parameters of DEGs. Figure 10 describes the dimensionless dissipated energy due to leakage current, *W*_leak_, the dimensionless harvested electrical energy, *E*_density_, the dimensionless mechanical energy, *W*_mech_, and the electromechanical conversion efficiency, *η*_c_, as functions of the capacitance ratio, *m*, and the voltage, Φ_H_. As shown in Figure 10a, as the voltage, Φ_H_, and the capacitance ratio, *m*, increase, the dissipative energy due to the leakage current can increase. Compared with the input mechanical energy (Figure 10c) and the harvested electrical energy (Figure 10b), only a small amount of energy is dissipated by the leakage current, which is consistent with the experimental observation [21]. As shown in Figure 10b, as the capacitance ratio, *m*, increases, the harvested electrical energy can increase rapidly and then decrease. Thus, there is an optimal capacitance ratio, *m*, to maximize the harvested electrical energy for the prescribed voltage, Φ_H_. It can be seen that increasing the voltage, Φ_H_, contributes to improving the harvested electrical energy. In Figure 10c, as the voltage, Φ_H_, and the capacitance ratio, *m*, increase, the input mechanical energy increases. It can be noticed from Figure 10d that similarly to the variation in the harvested electrical energy, there is an optimal capacitance ratio, *m*, to maximize the conversion efficiency. Moreover, with the increase in the voltage, Φ_H_, the conversion efficiency increases. Therefore, increasing the voltage, Φ_H_, during the discharging process can improve both energy density and conversion efficiency of a DEG. An appropriate capacitance ratio, *m*, can ensure maximization of the energy density or conversion efficiency. In addition, as mentioned above, when the voltage, Φ_L_, at state B is set as 3000 V (*m* = 1.3), the energy density of the DEG is slightly higher than the experimental result [21]. However, compared with the conversion efficiency (30%) reported in the literature [21], the efficiency achieved in our simulation is up to 49%. Thus, the designed energy harvesting cycle can significantly improve the conversion efficiency and ensure that the energy density could not be reduced. Furthermore, by optimizing the energy harvesting cycle, the DEG can achieve the highest energy density of 846 J/kg with the conversion efficiency of 50%. The main reason for the obvious improvement of the efficiency is that the voltage between the electrodes during the discharging process (from state C to D) is improved, and that in the designed cycle, the DEG does not relax during the LT process. Also, the energy dissipation caused by energy harvesting circuits and friction in the experiment setup is not considered in the simulation, which leads to the high efficiency.

**Figure 11:**
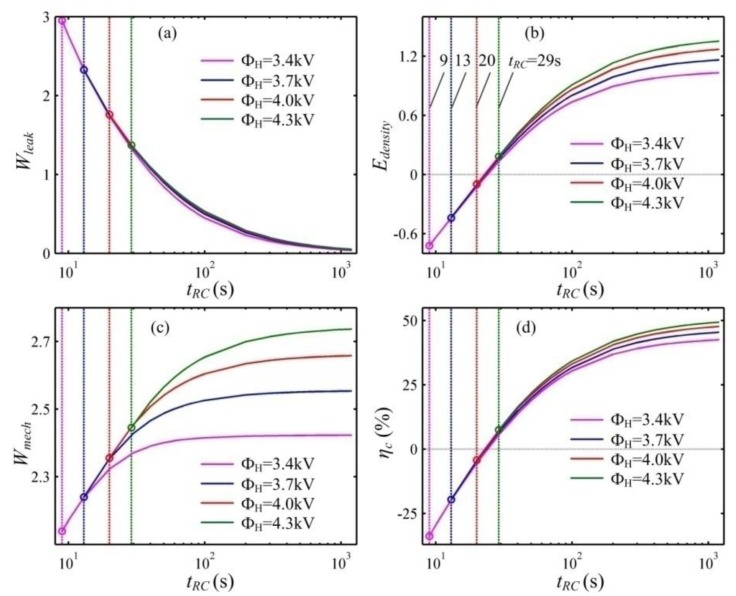
The influence of the R-C time constant on the performance of a DEG under different voltages, Φ_H_. (**a**) The dimensionless dissipated energy due to leakage current, *W**_leak_***, (**b**) the dimensionless energy density, *E_density_*, (**c**) the dimensionless mechanical energy, *W**_mech_***, and (**d**) the conversion efficiency, *η**_c_***. The capacitance ratio, *m* = 1.3, is fixed.

In addition, the leakage current can affect the energy harvesting performance of a DEG. When a DEG is subjected to the excessive leakage current, it can even waste electrical energy instead of generating it. We define the resistor-capacitor (R-C) time constant as the ratio between the permittivity and the conductivity of the DE, namely, *t*_RC_ = *ε*_0_*ε_r_*/*σ_c_*_0_, which is closely related to the leakage current. The DE materials exhibit a multiple R-C time constant varying from a few seconds to hundreds of seconds or even longer [2,20,30]. The smaller R-C time constant, the greater the leakage current is. Utilizing the developed theoretical framework, the energy harvesting performance of DEGs with different leakage currents can be evaluated by choosing different R-C time constants. Figure 11 describes the influence of the R-C time constant on the performance of a DEG under different voltages, Φ_H_. In this calculation, the capacitance ratio, *m* = 1.3, is set. To guarantee that the designed energy harvesting cycle can be executed, the R-C time constant should exceed the critical value. That is, the allowable area is on the right-hand side of the corresponding vertical line. As the voltage, Φ_H_, increases, the critical value increases. At the lower voltage, Φ_H_, if the R-C time constant is below the critical value, the voltage between the DE is still lower than Φ_H_ when the membrane is relaxed to the prescribed minimum stretch ratio. At the higher voltage, Φ_H_, when the R-C time constant is lower than the critical value, LT can occur before the voltage on the DE reaches Φ_H_. With the increase in the R-C time constant, the energy density and the conversion efficiency increase and then approach a constant. The dissipated energy due to the leakage current can decrease and then go to zero as the R-C time constant increases. Though the dissipated energy due to the leakage current can increase with the increase in the voltage, Φ_H_, increasing the voltage, Φ_H_, can still contribute to enhancing the energy density and the conversion efficiency. Moreover, as the R-C time constant increases, the enhancement effect of the voltage, Φ_H_, increases and then approaches saturation. In addition, it can be seen that when *t_RC_* < 24 s, both the energy density and the conversion efficiency are below zero, namely, the DEG can waste electrical energy instead of generating it. Thus, to guarantee that the DEG can generate electrical energy, the R-C time constant should exceed 24 s.

**Figure 12:**
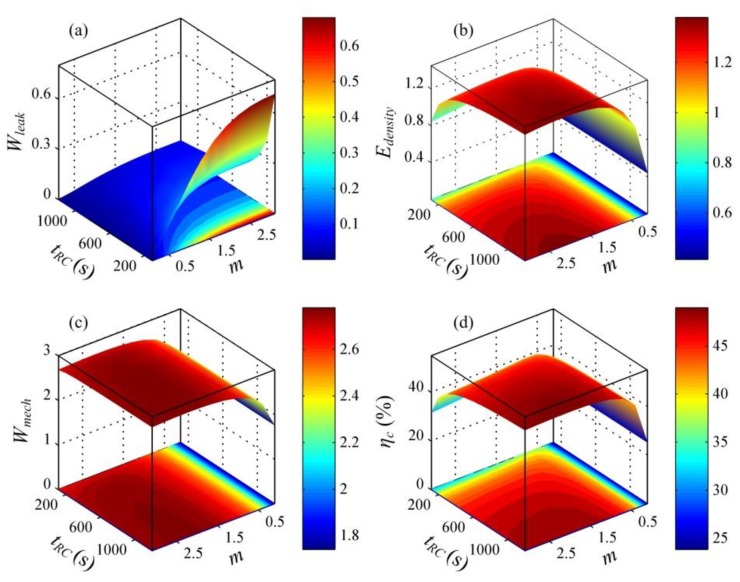
The influence of the R-C time constant, *t*_RC_, and the capacitance ratio, *m*, on the performance of a DEG. (**a**) The dimensionless dissipated energy due to leakage current, *W*_leak_, (**b**) the dimensionless energy density, *E*_density_, (**c**) the dimensionless mechanical energy, *W*_mech_, and (**d**) the conversion efficiency, *η*_c_. The voltage, Φ_H_ = 4300 V, is set.

Figure 12 depicts the influence of the R-C time constant, *t_RC_*, and the capacitance ratio, *m*, on the performance of a DEG. In this analysis, we set the voltage, Φ_H_ = 4300 V. As shown in Figure 12a, decreasing the R-C time constant, *t_RC_*, and increasing the capacitance ratio, *m*, can enhance the energy dissipation due to the leakage current. With the increase in the R-C time constant, *t_RC_*, the energy density and the conversion efficiency can increase (see Figure 12b,d)). As the capacitance ratio, *m*, increases, the energy density and the conversion efficiency can increase first and then decrease. There is an optimal capacitance ratio, *m*, to maximize the harvested electrical energy or the conversion efficiency for a prescribed R-C time constant, *t_RC_*. Moreover, as the R-C time constant, *t_RC_*, decreases, the optimal capacitance ratio, *m*, decreases and the maximum values of both the energy density and the conversion efficiency can decrease.

## 5. Conclusions

This paper explored a possible approach for improving both energy density and conversion efficiency of a dissipative DEG by considering the material viscosity and the leakage current. The theoretical framework of a dissipative DEG was developed. Based on the developed theoretical framework, the response of the DEG with the constant voltage cycle in which it can relax under the condition of no stress, i.e., during the LT process, was depicted, which showed good agreement with the existing experimental results. The results also showed that preventing the DEG from relaxing during the LT process in an energy harvesting cycle can improve the energy conversion performance. Then, we designed a novel energy harvesting cycle in which the DEG could not relax during the LT process and a corresponding energy harvesting circuit in which a transfer capacitor was utilized to store the charge transferred from the DEG, aimed at exploring a possible method for improving both energy density and conversion efficiency. Also, it is expected that compared with the existing experimental result, the conversion efficiency of the DEG with the novel energy harvesting cycle can be significantly improved and the corresponding energy density could not be reduced. On this basis, we investigated the influence of the transfer capacitor, the voltage between the electrodes during the discharging process, and the R-C time constant of the elastomer on the energy conversion performance of the DEG with the designed energy harvesting cycle. The simulation results showed that increasing the voltage during the discharging process was an effective method to improve both the energy density and the conversion efficiency, and that as the R-C time constant increased, the enhancement effect of the voltage increased and then approached saturation. After the voltage during the discharging process was set, choosing an optimal transfer capacitor ensured maximization of the energy density or the conversion efficiency. Also, with the increase in the R-C time constant, the optimal transfer capacitor can increase and the maximum values of both the energy density and the conversion efficiency can increase. These results and methods can help to provide guidance for the optimal design and assessment of DEGs.

## Figures and Tables

**Table 1:** The material parameters of VHB 4910 [2,25].

Material Parameters	Value
*J^α^*	110
*J^β^*	55
*χ*	0.5
*ε*_0_ (F/m)	8.85 × 10^−12^
*ε_r_*	3.5
*μ* (kPa)	600
*t*_v_ (s)	1
*E*_B_ (MV/m)	40
*σ*_c0_ (S/m)	3.23 × 10^−14^
